# A chinese medicine formula (kunbixiao granule) for female rheumatoid arthritis: Study protocol for a double-blind, randomized, placebo-controlled trial

**DOI:** 10.3389/fphar.2022.945565

**Published:** 2022-10-10

**Authors:** Yingying Wan, Jiaxi Yang, Tianyue Ma, Wenqian Wang, Haonan Wang, Wenting Sun, Wanting Ye, Lin Yang, Qiuai Kou

**Affiliations:** ^1^ Xiyuan Hospital of China Academy of Chinese Medical Sciences, Beijing, China; ^2^ Graduate School of Beijing, Beijing University of Chinese Medicine, Beijing, China

**Keywords:** rheumatoid arthritis, female, randomized controlled trial, double-blind, placebo, Chinese medicine, protocol

## Abstract

**Introduction:** Rheumatoid arthritis (RA) is a chronic autoimmune disease affecting females more than males. Clinical symptoms, disease activity and comorbidities are more severe in females. Moreover, the choice of treatment for females is limited during childbearing age due to the side effects of current drugs. Therefore, developing novel and safer drugs for females is urgently needed. Kunbixiao granules (KBXG), a Chinese medicine formula, has been applied to treat female RA patients in our center as a complementary therapy. However, there is insufficient evidence for its effect. Therefore, we aim to conduct a randomized, controlled, double-blind clinical trial to confirm the efficacy and safety of KBXG for the treatment of female RA.

**Methods:** This study is a single-center, double-blind, randomized, parallel group, placebo-controlled clinical trial. A total of 90 female RA patients with Disease Activity Score for 28 joints (DAS28) > 3.2 will be enrolled. They will be randomly assigned to receive either KBXG or placebo for 12 weeks. The change in DAS28 based on C-reactive protein (DAS28-CRP) and the Clinical Disease Activity Index (CDAI) are the primary outcomes. The secondary outcomes include a rate of achieving 20%, 50% and 70% improvement in the American College Rheumatology criteria (ACR20, ACR50, ACR70), TCM syndrome score, visual analogue scale (VAS), average hands grip strength, the consumption of concomitant medication, Hospital Anxiety and Depression Scale (HADS), lumbar spine bone mineral density (L-BMD) and 7-joint ultrasound score (US7). Any adverse events will also be recorded.

**Discussion:** This trial will provide evidence of KBXG in reducing disease activity, and improving clinical symptoms and quality of life of female RA patients. The long-term effects of KBXG on female RA patients still needs a further follow-up.

## Introduction

Rheumatoid arthritis (RA) is a chronic, progressive and autoimmune disease characterized by tissue damage, functional disorder, serious disability and premature mortality (Otón and Carmona, 2019; Firestein et al., 2020). A recent epidemiologic study showed RA affected about 1% of the world’s population (McInnes and Schett, 2017). The morbidity of RA is highest at the age of 50, with female patients as the main population (van der Woude and van der Helm-van Mil, 2018). Women are three to four times more prone to develop the disease than men, with data demonstrating a progressive increase in the incidence of RA in females during the last decade (Myasoedova et al., 2020). Meanwhile, RA seems to be more severe in women with higher disease activity, lower rates of remission and drug survival (Favalli et al., 2019; Maynard et al., 2020). More frequent comorbidities in female RA, such as depression, osteoporosis and fibromyalgia, also may impact treatment choice and outcomes (Albrecht, 2014).

At present, the treatments of RA mainly include non-steroidal anti-inflammatory drugs (NSAIDs), glucocorticoids, conventional synthetic disease-modifying anti-rheumatic drugs (csDMARDs) and biologic disease-modifying anti-rheumatic drugs (bDMARDs) (Singh et al., 2016). These drugs have multiple side effects, such as hepatorenal damage, peptic ulcer bleeding, cardiovascular complications, infection, and risk of carcinoma (Zamanpoor, 2019). Moreover, the use of these conventional drugs for treating female RA may be conditioned during childbearing age, pregnancy, and lactation (Ngian et al., 2016; Abbasi et al., 2019). Thus, it is necessary for exploring novel and safer therapeutic strategies for female patients of RA.

Traditional Chinese Medicine (TCM) has shown advantages in the treatment of RA. Chinese medicine formula monotherapy or combined with Western medicine had better performance on improving clinical symptoms, preventing the disease progression, reducing serum rheumatoid factor and anti-cyclic citrullinated peptide antibodies, and improving the bone mineral density of RA than Western medicine monotherapy (Cai, et al., 2018; Tang, et al., 2021; Zhang et al., 2022). But to date, there is no TCM clinical trial for RA in women. According to TCM theory, the Qi-blood deficiency and the toxin-heat blocking joints are the most common pathogenesis for female RA. KBXG, as a Chinese medicine formula, is composed of two traditional well-known formulas Simiao Yong’an decoction and Siwu decoction. It has been used in treating female RA patients in our center for decades. We have examined the efficacy of KBXG on collagen-induced arthritis (CIA) rats. The results showed that KBXG demonstrated favourable effect on inhibiting inflammation, synovial hyperplasia and joint destruction of CIA rats. (Ma et al., 2022). Additionally, our previous clinical study confirmed that KBXG could significantly improve clinical symptoms and reduce disease activity without any adverse events (AEs) (Niu, 2020). Nevertheless, there is no reliable evidence from evidence-based trials to popularize its use for treating female RA. Therefore, we conduct a well-designed, randomized double-blind controlled clinical trial to confirm the efficacy and safety of KBXG for the treatment of female RA.

## Methods

### Trial design

**FIGURE 1:**
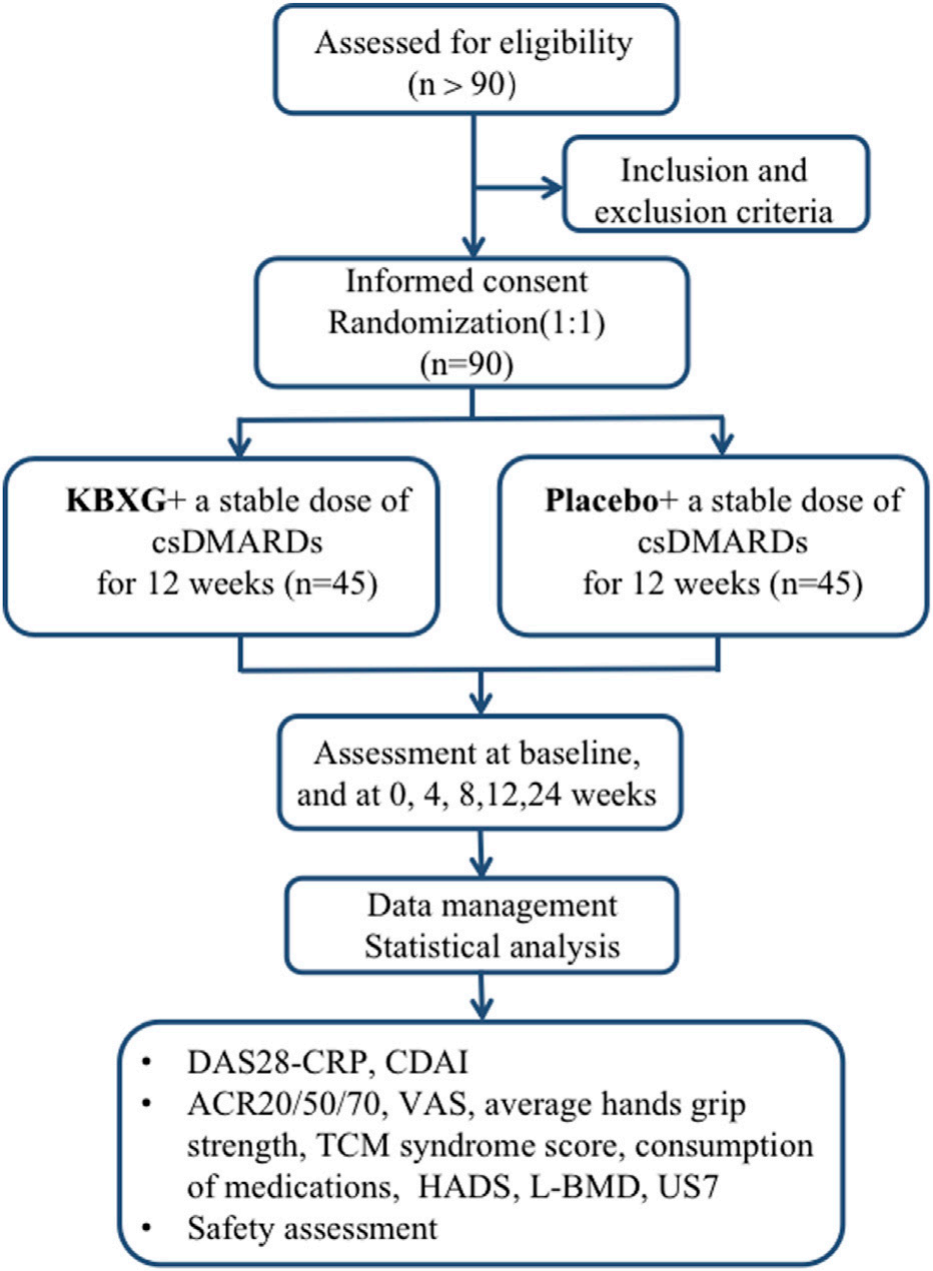
Study flowchart.

This is a single-center, randomized, double-blind, parallel group, placebo-controlled clinical trial. The trial will be conducted in the Xiyuan Hospital of China Academy of Chinese Medical Sciences. A total of 90 eligible female participants will be randomized into the experimental group (KBXG) or the control group (placebo) with a 1:1 ratio. Two groups will continue their original treatment regimen during the trial. The duration of the intervention is 12 weeks, and follow-up is 12 weeks. Study visits will take place at baseline and at 4, 8, 12, 24 weeks. Every patient will be asked to visit within 3 days of the given time point. All participants will be required to sign the written informed consent before randomization. This protocol was compiled in line with the Standard Protocol Items: Recommendations for Interventional Trials (SPIRIT) Guidelines (Chan et al., 2013), which is presented in the Supplementary Material S1. The flowchart of this trial is presented in Figure 1.

### Participants

90 female participants will be recruited at Xiyuan Hospital *via* putting up posters in the hospital areas. A rheumatologist will be responsible for screening participants and assessing whether they meet with the inclusion criteria. A research assistant will provide the participants with the informed consent form.

#### Diagnostic criteria

##### Diagnostic criteria for RA

In accordance with the classification criteria of RA proposed by ACR and European League against Rheumatism (EULAR) in 2010, participants with a score of 6 or above will be diagnosed with RA (Kay and Upchurch, 2012).

##### Diagnostic criteria for active RA

DAS28 > 3.2. DAS28, a commonly accepted measure for disease activity (van Riel and Renskers, 2016). According to EULAR diagnostic criteria for RA activity, 2.6 < DAS28 ≤ 3.2, 3.2 < DAS28 ≤ 5.1, and DAS28 > 5.1 represent low, moderate, and high disease activity, respectively (Fransen et al., 2004). DAS28 > 3.2 means the disease is active.

##### Diagnostic criteria for TCM syndrome differentiation

Patients have at least two of the primary symptoms, or one of the primary symptoms and more than two of the secondary symptoms, indispensable tongue and pulse listed in the following to be diagnosed with the Qi-blood deficiency, toxin-heat blocking joints syndrome.

1). Primary signs and symptoms: joint pain; joint swelling; morning stiffness; activity limitation.
2). Secondary signs and symptoms: redness of joint; local joint fever; fatigue; thirsty upset; dry stools and yellow urine.
3). Tongue and pulse: red tongue with yellow greasy or thin yellow fur, rapid and slippery pulse or soft pulse.

#### Inclusion criteria

Participants meeting the following criteria will be included:

1) Females aged 18–65 years old.
2) Patients who meet the diagnostic criteria for RA, meet the diagnostic criteria for active RA, and meet the Qi-blood deficiency, toxin-heat blocking joints syndrome.
3) A duration of RA for at least 3 months prior to enrollment.
4) Patients who take only one kind of csDMARDs (methotrexate at a dose of 15–20 mg per week at least or leflunomide at a dose of 20 mg per day) were given a stable dose for at least 12 weeks before screening and remained at the same dose for the rest of the treatment.
5) Patients who volunteer to participate in the trial and sign the informed consent form.

#### Exclusion criteria

Participants with any of the following criteria will be excluded:

1) Patients who are pregnant, lactating, or plan to become pregnant.
2) Patients with serious primary diseases involving in cardiovascular, liver, kidney, brain, endocrine and hematopoietic system, and psychiatric disorders.
3) Patients with severe chronic infection and carcinoma.
4) Patients with other rheumatic diseases including but not limited to myositis, systemic lupus erythematosus and sjogren’s syndrome.
5) Patients who are allergic or sensitized to the test drugs.
6) Patients who have changed their dose within 2 weeks of screening or have newly taken NSAIDs within 4 weeks of screening.
7) Patients who have received oral corticosteroids of more 10 mg per day, have newly taken within 4 weeks, or have changed their dose within 2 weeks of screening.
8) Patients who have received bDMARDs or received two or more csDMARDs before screening.

### Interventions

Patients in the experimental group will receive KBXG. Patients in the control group will receive placebo granules as the comparator. Two groups will continue their original treatment regimen during the trial. Both Kunbixiao granules and placebo granules will be provided by the Chinese Medicine Preparation Department, Xiyuan Hospital of China Academy of Chinese Medical Sciences. According to the quality control analysis, the quality of KBXG should meet the Chinese Medicine Standards of the State Food and Drug Administration. The herbal components of kunbixiao are shown in Table 1. Placebo granules are composed of 95% maltodextrin and 5% KBXG to achieve an odor, color, taste, and texture comparable to KBXG. The details are presented in the Supplementary Material S2. KBXG or placebo granules will be taken orally twice daily dissolving in warm water for 12 weeks. The methods and results on the quality control of KBXG and placebo granules are available in the Supplementary Material S3.

**TABLE 1:** The components of the Chinese herbal formula Kunbixiao.

Herbal name	Dosage(g)	Produced from
*Lonicera japonica* Thunb. [Caprifoliaceae]	30	Dried flower bud
Rehmannia glutinosa (Gaertn.) DC. [Orobanchaceae]	30	Dried root
Scrophularia ningpoensis Hemsl. [Scrophulariaceae]	20	Dried root
Paeonia lactiflora Pall. [Paeoniaceae]	15	Dried root
Cremastra appendiculata (D.Don) Makino [Orchidaceae]	10	Dried pseudobulb
Angelica sinensis (Oliv.) Diels [Apiaceae]	20	Dried root
Conioselinum anthriscoides ‘Chuanxiong’ [Apiaceae]	10	Dried rhizome
Codonopsis pilosula (Franch.) Nannf. [Campanulaceae]	30	Dried root
*Glycyrrhiza* glabra L. [Fabaceae]	10	Dried root and rhizome
Microsorum scolopendria (Burm.f.) Copel. [Polypodiaceae]	3	Dried body
Sinomenium acutum (Thunb.) Rehder & E.H.Wilson [Menispermaceae]	15	Dried rattan
Pyrola calliantha Andres [Ericaceae]	15	Dried herba

Participants are required to return both the opened and unopened drug and package. Counseling will be provided by rheumatologist about management in RA. Participants will be considered as dropouts if they fail to complete the study for various reasons or use prohibited drugs.

### Concomitant medications

#### Permitted

In this trial, patients taking only one kind of csDMARDs should be selected. CsDMARDs having been taken for more than 3 months with stable dose will be allowed and the dose cannot be changed during the trial. Patients who have taken NSAIDs and/or oral corticosteroid (less than 10 mg/day) with stable dose for at least 4 weeks will be permitted and any change of dose will be recorded. Notably, acetaminophen may be used if necessary (for patients with VAS for pain ≥6 cm) during the trial, and it will be recorded.

#### Prohibited

It includes medicines that can affect the primary outcome, such as 1) Chinese medicine with the same or similar function as KBXG, 2) corticosteroid injections, 3) Oral corticosteroid/NSAIDs administered for other diseases, and 4) bDMARDs, cell proliferation inhibitors, and/or immunosuppressants.

### Outcomes and measurements

#### Primary outcome

The primary outcomes are the change in DAS28-CRP and CDAI at every visit.

The DAS28-CRP is widely used as an indicator of RA disease activity and response to treatment, and clinical trials have used DAS28 to assess treatment effect (Gardiner et al., 2005; Jain et al., 2021; Zhang et al., 2022). A change of ≥1.2 score is considered clinically significant difference (Van Gestel et al., 1998). The DAS28-CRP is calculated as DAS28-CRP = 0.56*sqrt (TJC28) + 0.28*sqrt (SJC28) + 0.36*ln (CRP+1) + 0.014*VAS +0.96 (van Riel, 2014).

The CDAI is calculated as CDAI = TJC28 + SJC28 + overall disease activity on a 0–10 VAS completed by the patient + overall disease activity on a 0–10 VAS completed by the physician (Aletaha et al., 2005). High disease activity is defined as a CDAI >22, moderate activity as a CDAI >10 and ≤22, low activity as a CDAI >2.8 and ≤10, and remission as a CDAI ≤2.8.

#### Secondary outcomes

Secondary outcomes are the following:

1) The rate of achieving ACR20, ACR50, ACR70 (Felson and LaValley, 2014). Measurement will be performed at every visit. Briefly, ACR20/ACR50/ACR70 is defined as a more than 20%/50%/70% improvement respectively in both tender joint count (TJC) and swollen joint count (SJC). Patients should simultaneously have a more than 20%/50%/70% improvement respectively in at least 3 items as follow: patient’s assessment of pain on a VAS, patient’s global assessment of disease activity, physician’s global assessment of disease activity, HAQ-DI and CRP level.
2) Changes in TCM syndrome score. It will be evaluated based on the grading scales of RA symptoms required in the Guiding Principles of Clinical Research of New Chinese Medicine Treating RA (2002 Edition) issued by the National Medical Products Administration of China at every visit. The scores and details are presented in the Supplementary Material S4.
3) Changes in VAS. Pain will be evaluated using a VAS at every visit according to a former study (Faiz, 2014).
4) Changes in average hands grip strength. Measurement will be performed at every visit. The Jamar dynamometer is used to assess the hand grip strength (Higgins et al., 2018). For each strength test, three times of measurements should be done for each hand, and the mean score of both hands is recorded.
5) Changes in HADS. Anxiety and depression will be evaluated at every visit according to a former study (Herrero et al., 2003).
6) Changes in lumbar spine bone mineral density (L-BMD) to assess osteoporosis. Measurement will be performed at baseline and week 24.
7) Changes in 7-joint ultrasound score (US7). Measurement will be performed at baseline and week 12, 24. It determines the severity of synovitis, synovial hyperplasia and bone erosion of 7 joints through color Doppler ultrasound semi-quantitative assessment (Backhaus et al., 2009). The scores and details are presented in the Supplementary Material S5.
8) Consumption of concomitant medications. We will record any changes in the dose of NSAIDs or oral corticosteroids in the case report form (CRF). The drug name, total daily dose, and single dose will be record at every visit.

### Safety assessment

The interventions will be carried out under the guidance of a rheumatologist. The herbs in the KBXG are safe in accordance with the recommended amount in Chinese Pharmacopoeia. We will closely observe the physical conditions and subjective description of the participants. Blood, urine and stool routine analysis as well as liver and kidney function tests will be performed before enrollment and at 4, 8, and 12 weeks after enrollment. All adverse events will be recorded on the CRF and reported to the principal investigator. Any severe adverse events will be reported to the Ethics Committee of Xiyuan Hospital of China Academy of Chinese Medical Sciences within 24 h. The committee of medical experts will determine whether adverse events are related to the KBXG. Xiyuan Hospital of China Academy of Chinese Medical Sciences will cover the cost of treatment and the corresponding economic compensation for the damage related to the trial. Participants have the right to withdraw their consent to participate in the study at any time for any reason. Other unintended effects of study interventions and study conduct will also be recorded and reported.

### Participant timeline

The schedule of enrollment, interventions, and assessments is as shown in Table 2.

**TABLE 2:** A standard protocol items: recommendation for interventions for trials (SPIRIT).

	Study period						
	Enrollment	Allocation		Post-allocation			Follow up
Time point	- Day 3	Day 0	Week 0	Week 4	Week 8	Week 12	Week 24
Enrolment							
Eligibility screen	X						
Informed consent	X						
Allocation		X					
Interventions							
KBXG			X	X	X	X	
Placebo			X	X	X	X	
Assessments							
DAS28-CRP	X			X	X	X	X
CDAI			X	X	X	X	
ACR20/50/70			X	X	X	X	
TCM syndrome score			X	X	X	X	X
VAS			X	X	X	X	X
Average hands grip strength			X	X	X	X	X
HADS			X	X	X	X	X
L-BMD			X				X
US7			X			X	X
Consumption of concomitant medications	X		X	X	X	X	
ESR, CRP	X			X	X	X	X
Blood routine	X					X	X
Urine routine	X					X	X
Stool routine	X					X	X
Liver and kidney function	X					X	X
AEs			X	X	X	X	

### Sample size

The sample size is calculated based on the primary endpoint (the changes in DAS28-CRP). According to the previous exploratory clinical trial (unpublished), the effect size of the treatment group was 83.9%, with a power of 0.9 and alpha of 0.05, a total of 68 female patients should be included to determine an increase of 15% in disease activity improvement between the KBXG and placebo groups. To account for a 20% loss to follow-up, at least 85 female patients will be required in this trial. Thus, a total of 90 female patients (45 female patients in each group) are planned to be enrolled.

### Randomization and allocation

The random sequence will be generated by an independent statistician from the Good Clinical Practice (GCP) Centre of Xiyuan Hospital of China Academy of Chinese Medical Sciences. Participants will be randomly allocated into either the treatment or placebo group, at a ratio of 1:1. Patients in the treatment group (*n* = 45) will receive the treatment of KBXG, while patients in the placebo group (*n* = 45) will receive placebo granules, that are identical in appearance to the KBXG. The KBXG and placebo with same packaging will be labeled according to the random number table. The random sequence will be kept in opaque sealed envelopes with consecutive numbers. These envelopes will be prepared by the GCP Centre. A rheumatologist will enroll participants. A research assistant will open the same numbered envelope based on the randomization enrollment number when an eligible participant can be included in the trial. Then, the research assistant will assign the drugs to the participant according to the sequence.

### Blinding

This is a double-blind trial in which patients, researchers, outcome assessors, and statistician are all blinded. Assignment of interventions will only be unblinded after database lock. Unblinding is permissible in emergency situations when researchers believe that it is necessary to perform any action for patient’s safety. The details of unblinding will be recorded.

### Data quality control

All researchers will receive professional training to understand the protocol. The researchers will obey to guidelines of GCP to ensure the safety, blinding and data quality. A specially trained researcher will carry out a quality control assessment every month to ensure the high-quality of this study.

### Data collection and management

Researchers are responsible for the collection of baseline information and trial data. The researchers will ensure that the study schedule is fully explained and participants are aware of the potential risks and benefits of the treatment before consent is obtained. Participants will receive phone calls before each visit to remind them of their time. Two independent investigators will double - checked all CRF. Data from the CRF will be inputted into the SPSS by the two data administrator. Once the trial is completed, we will perform a blind review to confirm the dataset. The final data will either be kept in a secure and lock-protected location.

### Statistical methods

Data analysis will be completed using SPSS 23.0, according to the intention-to-treat analysis and the per-protocol analysis. Baseline analyses will be performed using analysis of variance or non-parametric tests for continuous variables, and chi-squared or Fisher’s exact tests for categorical variables, respectively. Changes from baseline to post-treatment in DAS28-CRP, CDAI, TCM syndrome scores, VAS, average hands grip strength, HADS, L-BDM and US7 will be analyzed. The results of the two groups will be analyzed using analysis of covariance with associated 95% confidence intervals. The rates of participants achieving good responses on ACR will be compared. A two-sided *p* value of < or = 0.05 is considered statistically significant. Multiple imputations will be used to handle any missing data. For safety, the incidence of AEs will be analyzed and compared. The details of each AEs will be analyzed.

Participants with poor adherence, compliance of experimental drug <80%, or those no longer receiving medication and undergoing testing during the study will be included in the intention-to-treat analysis and excluded from the per-protocol analysis. Multiple imputation will be used for missing data.

### Data monitoring, harms and auditing

The Data Monitoring Committee (DMC) of Xiyuan Hospital of China Academy of Chinese Medical Sciences will meet once half a year to review trial conduct. The committee will conduct primary outcome interim analyses to oversee the safety and efficacy of the study when it reaches 50% of the recruitment. Besides, the committee will assess the status and quality of the study and make recommendations to continue or terminate the study. KBXG is relatively safe, and AEs are mild. Every patient could report any AEs to the researchers *via* phone and the researchers would immediately take action.

### Ethics and dissemination

Protocol version: v3.0 was finished on 1 May 2021. This trial has been approved by the Ethics Committee of Xiyuan Hospital of China Academy of Chinese Medical Sciences (2021XLA014-3) and will be conducted under the supervision of the Ethics Committee. Any modifications to the protocol regarding trial design, eligibility criteria, sample sizes, or significant changes in the study will initially require agreement from the Ethics Committee. Participants will sign the informed consent forms at the baseline and their personal information will remain strictly confidential. Results will be published in a peer-reviewed academic journal. The full protocol, participant-level data, and statistical code are available from the corresponding author upon reasonable request.

## Discussion

RA is a chronic inflammatory disease with high morbidity and mortality. It is reported that the life expectancy of RA patients is usually 6–11 years less than that of normal people. Notably, the majority of them are females (Cush, 2021). Moreover, disease activity, radiographic damage and physical disability of female RA patients are more severe than that of male (Kuiper et al., 2001). Recurrent joint pain, progressive joint deformation and even disability have a substantial effect on female patients’ physical and mental health. Due to the side effects of conventional drugs, the choices of treatment of female RA patients are also conditioned during childbearing age, pregnancy, and lactation. Thus, the optimal treatment regimen for female RA patients should be carefully considered.

As a complementary and alternative medicine, TCM is widely used in RA treatment in China, which has shown the effects of reducing disease activity, improving clinical symptoms, and enhancing the quality of life. In our previous small sample exploratory clinical study, we found that compared with hydroxychloroquine sulfate, KBXG significantly improved clinical symptoms and joint function, reduced disease activity and the level of inflammation factors of female RA patients. At the same time, no adverse events (AEs) occurred (Niu, 2020). Therefore, it is necessary to conduct standardized randomized controlled clinical trials to confirm the efficacy and safety of KBXG.

RA can be characterized by the accumulation of inflammatory cytokines in the synovial joint, resulting in pannus formation, cartilage degradation and bone destruction. In particular, the formation of new microvessels within the synovium, is known as major features of blood vessels in RA (Semerano et al., 2011). These pathological changes in RA are similar to toxin-heat pathogenic factors, which is a traditional pattern-identification diagnosis in TCM. Toxin-heat can cause disease activity. For female RA patients, physiological activities like menstruation, pregnancy, parturition and breastfeeding, can result in a deficiency of Qi and blood, further aggravating toxin-heat accumulation and causing joint swelling and pain. KBXG, consisting of two traditional well-known formulas Simiao Yong’an decoction and Siwu decoction, has been used to benefit Qi and blood, and remove toxin-heat (Fang and Kou, 2018). Based on this theory, KBXG is more suitable for female patients with RA. Moreover, Simiao Yong’an decoction has a good effect on RA patients and also inhibits inflammatory cytokines and synovial hyperplasia in CIA rats (Zhao and Qiu, 2011; Wang et al., 2015). Siwu decoction can significantly improve the clinical symptoms of RA patients as a complementary therapy (Liu et al., 2017). Thus, we developed KBXG to treat the toxin-heat syndrome of female RA.

Based on the current evidence, we designed this double-blinded randomized controlled clinical trial. To our knowledge, this is the first randomized controlled trial that explores the efficacy and safety of KBXG for the treatment of female RA patients associated with the toxin-heat syndrome. The success of our trial will provide a high-quality evidence-based basis that KBXG is safe and efficacious for treating female RA patients. Besides, there will be preliminary evidence indicating that KBXG can improve the quality of life and depression state of female RA patients.

The study also has certain limitations. The single-center study may not extrapolate the results to other ethnic groups or regions. Sample size and treatment duration are calculated based on our previous exploratory study, which may lead to an overestimation of the efficacy of KBXG. Longer follow-up is needed to observe the effect of KBXG on the clinical prognosis such as joint dysfunction and disability in female RA patients.

## Data Availability

The raw data supporting the conclusion of this article will be made available by the authors, without undue reservation.
