# Corrigendum: A Chinese medicine formula (kunbixiao granule) for female rheumatoid arthritis: Study protocol for a double-blind, randomized, placebo-controlled trial

**DOI:** 10.3389/fphar.2022.1074679

**Published:** 2022-11-03

**Authors:** Yingying Wan, Jiaxi Yang, Tianyue Ma, Wenqian Wang, Haonan Wang, Wenting Sun, Wanting Ye, Lin Yang, Qiuai Kou

**Affiliations:** ^1^ Xiyuan Hospital of China Academy of Chinese Medical Sciences, Beijing, China; ^2^ Beijing University of Chinese Medicine, Beijing, China

**Keywords:** rheumatoid arthritis, female, randomized controlled trial, double-blind, placebo, Chinese medicine, protocol

In the published article, Jianxun Ren was erroneously included. The corrected author list and correspondence information appear below. Additionally, the Author contributions section has been revised to reflect this amendment.

Yingying Wan^1†^, Jiaxi Yang^2†^, Tianyue Ma^2^, Wenqian Wang^2^, Haonan Wang^1^, Wenting Sun^1^, Wanting Ye^2^, Lin Yang^1^ and Qiuai Kou^1^*

**Correspondence**: Qiuai Kou, kouqiuai@163.com

**Author Contributions:** QK, YW, and JY contributed to the study design. YW drafted the manuscript, and prepared the additional files. JY was responsible for obtaining ethics approval. TM, WW, HW, WY, and WS devised and discussed the study design. LY analyzed the main components of the Chinese medicine. All authors have read and agreed with the final manuscript.

The authors apologize for this error and state that this does not change the scientific conclusions of the article in any way. The original article has been updated.

