# Supplementary material for: A chinese medicine formula (kunbixiao granule) for female rheumatoid arthritis: Study protocol for a double-blind, randomized, placebo-controlled trial
Source: Front Pharmacol. 2022 Oct 10;13:945565. doi: 10.3389/fphar.2022.945565 (PMC9592086; doi:10.3389/fphar.2022.945565)
Supplement: Supplementary file 2 [file Table2.DOCX]

**1. The components of the placebo granules**

| Components | Proportion(%) |
| --- | --- |
| Maltodextrin | 95% |
| Caramel color |  |
| Sunset yellow |  |
| Bitterant |  |
| Kunbixiao granules | 5% |

**2. The components of the Kunbixiao**

| Components | Dosage (g) |
| --- | --- |
| Lonicera japonica Thunb. [Caprifoliaceae] | 30 |
| Rehmannia glutinosa (Gaertn.) DC. [Orobanchaceae] | 30 |
| Scrophularia ningpoensis Hemsl. [Scrophulariaceae] | 20 |
| Paeonia lactiflora Pall. [Paeoniaceae] | 15 |
| Cremastra appendiculata (D.Don) Makino [Orchidaceae] | 10 |
| Angelica sinensis (Oliv.) Diels [Apiaceae] | 20 |
| Conioselinum anthriscoides 'Chuanxiong' [Apiaceae] | 10 |
| Codonopsis pilosula (Franch.) Nannf. [Campanulaceae] | 30 |
| Glycyrrhiza glabra L. [Fabaceae] | 10 |
| Microsorum scolopendria (Burm.f.) Copel. [Polypodiaceae] | 3 |
| Sinomenium acutum (Thunb.) Rehder & E.H.Wilson [Menispermaceae] | 15 |
| Pyrola calliantha Andres [Ericaceae] | 15 |
