# Supplementary material for: A chinese medicine formula (kunbixiao granule) for female rheumatoid arthritis: Study protocol for a double-blind, randomized, placebo-controlled trial
Source: Front Pharmacol. 2022 Oct 10;13:945565. doi: 10.3389/fphar.2022.945565 (PMC9592086; doi:10.3389/fphar.2022.945565)
Supplement: Supplementary file 3 [file Table5.DOCX]

**Table S5** 7-joint ultrasound score

| **Score** | **Synovitis** | **Tenosynovitis** | **Bone erosions** | **Color Doppler flow signal** |
| --- | --- | --- | --- | --- |
| 0 | No synovial thickening | No hypoechoic thickened tissue | No | No intraarticular color signal |
| 1 | Mild: a small hypoechoic/anechoic line beneath the joint capsule | A hypoechoic thickened tissue with or without fluid and Doppler flow signal within the tendon sheath | Present (an interruption of the bone surface in 2 perpendicular planes) | Up to three color signals or two single and one confluent signal in the intraarticular area |
| 2 | Moderate: the joint capsule is elevated parallel to the joint area |  |  | Greater than grade 1 to <50% of the intraarticular area filled with color signals |
| 3 | Severe: a strong distension of the joint capsule |  |  | ≥50% of the intraarticular area filled with color signals |
