# Supplementary material for: A chinese medicine formula (kunbixiao granule) for female rheumatoid arthritis: Study protocol for a double-blind, randomized, placebo-controlled trial
Source: Front Pharmacol. 2022 Oct 10;13:945565. doi: 10.3389/fphar.2022.945565 (PMC9592086; doi:10.3389/fphar.2022.945565)
Supplement: Supplementary file 4 [file Table3.DOCX]

**UPLC-Q-TOF-MS analysis of KBXG and placebo granules**

**1. Preparation of the KBXG solution**

We weighed 10.3g of the KBXG and put them into a conical flask, subsequently adding 30ml of 70% methanol and processing with ultrasonic for 30 minutes. An appropriate amount of extract was taken and filtered with 0.22μm filter membrane. An appropriate amount of filtrate was diluted 7 times with 70% methanol, mixed with vortex machine, and then centrifuged at 12000r/min for 5 minutes to obtain the supernatant. Furthermore, the supernatant was then injected into the Waters Acquity UPLC H-CLASS system (Waters, USA).

**2. Preparation of the placebo granules solution**

We weighed 10.3g of the placebo granules and put them into a conical flask, subsequently adding 30ml of 70% methanol and processing with ultrasonic for 30 minutes. An appropriate amount of extract was taken and filtered with 0.22μm filter membrane. An appropriate amount of filtrate was diluted 7 times with 70% methanol, mixed with vortex machine, and then centrifuged at 12000r/min for 5 minutes to obtain the supernatant. Furthermore, the supernatant was then injected into the Waters Acquity UPLC H-CLASS system (Waters, USA).

**3. Chromatographic and mass spectrometry conditions**

The chromatographic separation was performed using a ACQUITY UPLC HSS T3 column (2.1×100mm, 1.8μm) at 35℃. 0.1% formic acid acetonitrile solution (A) and 0.1% formic acid water solution (B) were used for mobile phase analysis with a flow rate of 0.4mL/min. The injection volume was 2μl. Q-TOF-MS analysis was carried out in positive and negative ion modes on Xevo G2-S Quadrupole Time-of-Flight Mass Spectrometer equipped with a water electrospray ionization interface (Waters, USA). The electrospray ionization source (ESI) was scanned in positive and negative ion mode. The scanning range was m/z 100 - 1200.

**4. Experimental results**

We analyzed the main components of the KBXG and placebo granules using UPLC-Q-TOF-MS. Base peak intensity (BPI) chromatograms of the KBXG in negative ion mode and positive ion mode were shown in **Figures 1, 2**. The chemical structures of the main components were qualitatively identified using mass spectrometry software (MassLynx v4.1). Based on MS and MS/MS analysis results and existing reference materials, 14 chemical compounds were identified in the KBXG, including harpagide, cryptochlorogenic acid, chlorogenic acid, neochlorogenic acid, Isochlorogenic acid B, Isochlorogenic acid A, Isochlorogenic acid C, albiflorin, paeoniflorin, angoroside C and harpagoside in negative ion mode, as well as sinomenine, magnoflorine and hyperoside in positive ion mode. BPI chromatograms of the placebo granules in positive ion mode and negative ion mode were shown in **Figures 3**.

**Figure 1.** Base peak intensity (BPI) chromatogram of KBXG in negative ion mode (1: harpagide; 2: cryptochlorogenic acid; 3: chlorogenic acid; 4: neochlorogenic acid; 5: Isochlorogenic acid B; 6: Isochlorogenic acid A; 7: Isochlorogenic acid C; 8: albiflorin; 9: paeoniflorin; 10: angoroside C; 11: harpagoside)

**Figure 2.** BPI chromatogram of KBXG in positive ion mode (12: sinomenine; 13: magnoflorine; 14: Hyperoside)

**Figure 3.** (A) BPI chromatogram of placebo granules in positive ion mode. (B) BPI chromatogram of KBXG in positive ion mode. (C) BPI chromatogram of placebo granules in negative ion mode. (D) BPI chromatogram of KBXG in negative ion mode.
