# Supplementary material for: A chinese medicine formula (kunbixiao granule) for female rheumatoid arthritis: Study protocol for a double-blind, randomized, placebo-controlled trial
Source: Front Pharmacol. 2022 Oct 10;13:945565. doi: 10.3389/fphar.2022.945565 (PMC9592086; doi:10.3389/fphar.2022.945565)
Supplement: Supplementary file 5 [file Table4.DOCX]

**Tables S4** TCM syndrome score and detailed description of symptoms

| **symptoms** | | **None/0 score** | **Mild/2 score** | **Moderate/4 score** | **Severe/6 score** |
| --- | --- | --- | --- | --- | --- |
| Primary symptoms | Joint pain | No symptom | Slight, not influencing work and life | Heavy, influencing work and life | Severe, seriously influencing work and life, needing to take NSAIDs |
|  | Joint swelling | No symptom | Slight, visible skin texture and bone landmark | Heavy, almost invisible skin texture and bone landmark | Severe, disappeared skin texture and bone landmark |
|  | Morning stiffness | No symptom | Duration: < 30 minutes | Duration: > 30 minutes, <60 minutes | Duration: > 60 minutes |
|  | Activity limitation | No symptom | Slightly limited, with < 1/3 reduction in joint motion range | Significantly limited, with ≥1/3 reduction in joint motion range | Significantly limited, with ≥2/3 reduction in joint motion range |
|  |  | **None/0 score** | **Mild/1 score** | **Moderate/2 score** | **Severe/3 score** |
| Secondary symptoms | Local joint fever | No symptom | Joint touches hot, but patient does not feel hot | Joint touches hot, and patient feels hot | Joint touches hot and patient feels significantly hot |
|  | Joint tenderness | No symptom | Patient feels pain when pressed joint | Patient feels pain and frowns uncomfortably when pressed joint | Patient feels intolerable pain and pulls the hand or limbs back |
|  | Fever | No symptom | Temperature: 37.5℃-37.9℃ | Temperature: 38.0℃-38.9℃ | Temperature: >39℃ |
|  | Fatigue | No symptom | Occasionally | Frequently | Always |
|  | complexion | No symptom | Dull complexion | Dull, pale and/or yellow complexion | Significantly dull and unadorned complexion |
|  | Thirsty | No symptom | Occasionally | Frequently | Always |
|  | Sweating | No symptom | Occasionally | Frequently | Always |
